# Screening for neurocysticercosis in internationally adopted children: yield, cost and performance of serological tests, Italy, 2001 to 2016

**DOI:** 10.2807/1560-7917.ES.2018.23.40.1700709

**Published:** 2018-10-04

**Authors:** Lorenzo Zammarchi, Andrea Angheben, Teresa Fantoni, Elena Chiappini, Antonia Mantella, Luisa Galli, Valentina Marchese, Giorgio Zavarise, Zeno Bisoffi, Alessandro Bartoloni

**Affiliations:** 1 Department of Experimental and Clinical Medicine, University of Florence, Florence, Italy; 2 Infectious and Tropical Diseases Unit, Careggi University Hospital, Florence, Italy; 3 Centre for Tropical Diseases, Sacro Cuore – Don Calabria Hospital, Negrar, Italy; 4 Infectious Disease Unit, Meyer University Hospital, Department of Health Science, University of Florence, Florence, Italy; 5 Health Human Sciences School, Medicine and Surgery Degree Course, University of Florence, Florence, Italy; 6 Department of Pediatrics, Hospital Sacro Cuore – Don Calabria, Negrar, Italy; 7 University Department of Infectious and Tropical Diseases & WHO Collaborating Centre for TB/HIV and TB elimination, University of Brescia, Brescia, Italy

**Keywords:** cysticercosis, neurocysticercosis, screening, children, Italy, adopted

## Abstract

Neurocysticercosis (NCC) is one of the leading causes of epilepsy worldwide. The majority of cases in Europe are diagnosed in immigrants. Currently in Italy, routine serological screening for cysticercosis is recommended for internationally adopted children (IAC) coming from endemic countries. **Methods:** We retrospectively analyse the results of the serological screening for cysticercosis in IAC 16 years old or younger, attending two Italian third level paediatric clinics in 2001–16. **Results:** Of 2,973 children included in the study, 2,437 (82.0%) were screened by enzyme-linked immune electro transfer blot (EITB), 1,534 (51.6%) by ELISA, and 998 (33.6%) by both tests. The seroprevalence of cysticercosis ranged between 1.7% and 8.9% according to EITB and ELISA, respectively. Overall, 13 children were diagnosed with NCC accounting for a NCC frequency of 0.4% (95% confidence interval (CI): 0.2–0.6%). Among the 168 seropositive children, only seven (4.2%) were diagnosed with NCC. Of these children, three were asymptomatic and four presented epilepsy. Among seronegative children (n = 2,805), seven presented with neurological symptoms that lead to the diagnosis of NCC in six cases. The sensitivity, specificity, positive and negative predictive value for the diagnosis of NCC were 54.5%, 98.6%, 14.6%, 99.8% for EITB and 22.2%, 91.1%, 1.4%, 99.5% for ELISA. The yield of the screening programme was 437 NCC cases per 100,000. The number needed to screen to detect one NCC case was 228. The cost per NCC case detected was EUR 10,372. **Conclusion:** On the base of our findings we suggest the ongoing serological screening for cysticercosis to be discontinued, at least in Italy, until further evidence in support will be available.

## Introduction

Human cysticercosis is due to the invasion of tissues by the metacestode larval stage of *Taenia solium* [1]. The central nervous system is the most clinically relevant localisation of the parasite, which in this case causes neurocysticercosis (NCC) [1]. *T. solium* and NCC are currently endemic in large areas of Latin America, sub-Saharan Africa, and Asia, including the Indian subcontinent, most of south-east Asia, and China [1]. In the United States (US) and Western Europe, the disease is mainly imported [2,3]. The World Health Organization in 2017 reported that the total estimated number of people with NCC in endemic areas range between 2.56 million and 8.30 million [4]. In Western Europe, according to the latest systematic review available, only 275 cases of cysticercosis were reported in the period 1990–2015, of whom only 5% were suspected to be autochthonous [5].

NCC is the most frequent preventable cause of epilepsy in the developing world [6] being responsible for ca 30% of cases of epilepsy in low income countries [7,8]. More severe manifestations such as focal neurologic deficits, intracranial hypertension, cognitive decline and chronic meningitis are more rarely observed [1]. Children are less frequently affected by NCC than adults [9-11], probably due to mechanisms involved in disease acquisition and differences in the reactivity of the immune system against the parasite. However, NCC is responsible for a non-negligible portion of epilepsy cases in children as well. In Northern India, the reported NCC prevalence was 4.5% among children aged 1 to 14 years presenting to a tertiary care hospital with a first episode of seizure or acute focal neurological deficit [12]. According to another community-based study conducted in the city of Kolkata, India, the prevalence of NCC among patients aged 19 years or less with active epilepsy was 23.4% [13]. In Lima, Peru, NCC was found to be the main cause of partial seizure among preschool and school-aged children accessing a public hospital, accounting for 37% of cases [14]. According to large case series observed in Europe and in the US, paediatric patients account for 21–26% of NCC cases, the majority of them diagnosed in immigrant children or children who had travelled to endemic areas [15,16]. Internationally adopted children (IAC) represent a considerable and peculiar portion of immigrant children. Thousands of children are internationally adopted each year from lower income countries, especially in European countries, in the US and Canada [17]. Although children are declared healthy in their home countries, medical disorders are often missed, with diagnosis only occuring after adoption [18-21]. For these reasons, IAC usually undergo a comprehensive medical evaluation upon arrival in the host country, where frequently unsuspected disorders such as infections are identified [20,22].

In the period 2001–15, ca 3,000 IAC arrived in Italy each year (range ca 1,800–4,000). Of them, about 50% came from other European countries, ca 25% from the Americas, ca 15% from Asia and ca 10% from Africa (Supplement: Table 1). Upon arrival to Italy (usually within 3–4 months), IAC usually undergo a comprehensive medical evaluation according to a protocol suggested by the ‘National Working Group for the Migrant Child’ of the Italian Society of Paediatrics in one of the 19 dedicated reference centres, identified by the Italian Society of Paediatrics [23]. The medical screening protocol suggests to investigate the presence of several non-infectious and infectious diseases, including NCC. According to the latest version of the Italian Society of Paediatrics protocol issued in 2013, the medical evaluation of IAC includes a serological screening test for cysticercosis (the method of serological testing is not specified) for children coming from Asia and Latin America (including the Caribbean), while in previous versions that covered the study period, the serological screening was recommended for Asian and Latin American children, as well as for African ones [24-26]. Individuals with a positive test for cysticercosis undergo a full clinical assessment including, at least in the two paediatric reference centres participating in the current study, a brain Magnetic Resonance Imaging (MRI). Whereas the serology-based screening for NCC in IAC is widely used in Italy, and even in other countries such as France [27], there are few data on the yield, cost and performance of this strategy. The objective of this study was to evaluate the yield, cost and performance of a serological screening protocol for NCC in IAC.

## Methods

### Study design and population

This was an observational retrospective study. We included all IAC aged 16 years or less, who consecutively underwent a serological test for cysticercosis in a 16 year period (from 1 January 2001 to 31 December 2016) at two paediatric reference centres for IAC, namely the Infectious Diseases Unit, Meyer University Hospital, Department of Health Science, University of Florence, Florence, Italy (MY) and the Department of Paediatrics, Sacro Cuore – Don Calabria Hospital, Negrar, Italy (NE). During the study period, except for after 2013, when children from Africa were no longer recommended for screening, the protocol of the ‘National Working Group for the Migrant Child’ of the Italian Society of Paediatrics suggested that the test for cysticercosis should be offered to all IAC coming from Latin American, African, Asian countries. Of note, some IAC coming from Eastern European countries were sometimes screened for cysticercosis despite not being mentioned in the protocol. Likewise, after 2013, some children from Africa were occasionally tested. 

### Data source and data extraction

Epidemiological and clinical data (including some laboratory results of routine tests performed when the individuals accessed the centres) were extracted by consulting the clinical records. For every child, the following data were retrieved and collected using an electronic Excel database: age, sex, geographical area of the world and country of birth, date of visit, type of cysticercosis serological test performed and its result, absolute eosinophil count, result of stool parasitological test, presence or absence of neurological symptoms, diagnosis of NCC according to the diagnostic criteria proposed by Del Brutto et al. (in 2017) [28].

For individuals diagnosed with NCC according to the aforementioned criteria, detailed epidemiological, clinical, laboratory and radiology information to ascertain the presence or absence of each diagnostic criteria were checked and registered in an additional database. Supplement Table 2 reports Del Brutto et al. diagnostic criteria (2017 version) and their interpretation.

### Serological test for cysticercosis

At the MY, individuals were screened with a commercial enzyme-linked immune electro transfer blot (EITB) test on serum (Cysticercosis western blot IgG) produced by LDBIO Diagnostics (Lyon, France) throughout the study period (2001–16). The test was performed and interpreted according to the test manufacturer's instructions that varied over the years. In the period 2001–13, the presence of at least one of five specific bands (6–8kDa, 12kDa, 23–26kDa, 39kDa, 50–55kDa) was considered as a criterion of positivity. From 2014 onwards, the presence of at least two specific bands was required for considering a test positive [29].

At the NE centre, in the period 2001–4, serum samples were tested with an enzyme-linked immunosorbent assay (ELISA) test. In the period 2005–15 serum samples were tested in parallel with ELISA and EITB, while since 2016 only EITB was used. The ELISA test used was a commercial one (ELISA, Taenia Solium IgG, DRG International Inc, Springfield, US). The test was performed and interpreted according to the manufacturer's instruction. Absorbance equal to or greater than 0.3 (dilution 1:64 on 100 µL of serum) was considered as corresponding to a positive test result [30]. In the period 2005–6 the EITB used was Qualicode Cysticercosis kit (Immunetics Inc., Boston, Massachusetts, US), and the test was considered positive in presence of at least one of the specific bands (50 KDa, 42–39kDa, 24kDa, 21kDa, 18kDa, 14kDa, 13kDa) [31]. From 2007, the EITB produced by Immunetics was replaced by the EITB produced by LDBIO Diagnostics, interpreted according to the manufacturer's instruction (as for the MY centre). In 2016, persons evaluated in NE were tested only with the EITB. Table 1 summarises the serological tests for cysticercosis used during the study period in the two centres.

**Table 1:** Different serological tests for cysticercosis used in two paediatric reference centres for internationally adopted children ≤16 years old, Italy, 2001–2016

Period	Meyer University Hospital^a^	Sacro Cuore-Don Calabria Hospital^b^
2001–2004	EITB^c^	ELISA^d^
2005–2006	EITB^c^	ELISA^d^ + EITB^e^
2007–2015	EITB^c^	ELISA^d^ + EITB^c^
2016	EITB^c^	EITB^c^

EITB: enzyme-linked immune electro transfer blot; ELISA: enzyme-linked immunosorbent assay; US: United States.

^a^ Infectious Diseases Unit, Meyer University Hospital, Florence, Italy.

^b^ Department of Paediatrics, Sacro Cuore-Don Calabria Hospital, Negrar, Italy.

^c^ Cysticercosis Western blot IgG, LDBIO Diagnostics, Lyon, France.

^d^ Taenia Solium IgG, DRG International Inc, Springfield, US.

^e^ QualiCode Cysticercosis kit, Immunetics Inc., Boston, Massachusetts, US.

### Data analysis

For the analysis purpose, EITB tests performed with the different kits (Immunetics and LDBIO) were considered as one test. The following parameters were estimated: seroprevalence for cysticercosis according to the different serological tests used (ELISA and EITB), sex, age, country and world geographical area of birth; frequency of NCC (diagnosed according to the diagnostic criteria proposed by Del Brutto et al. in 2017, see Supplement: Table 2) according to sex, age, country and geographical area of birth; sensitivity, specificity, positive predictive value (PPV) and negative predictive value (NPV) of EITB and ELISA considering as gold standard the diagnosis of NCC according to the diagnostic criteria proposed by Del Brutto et al. in 2017; association (with chi-squared test) between positivity to ELISA or EITB and eosinophilia (> 450 eosinophils per µL) and diagnosis of other helminthic infections; association (with chi-squared test) between diagnosis of NCC according to the diagnostic criteria proposed by Del Brutto et al. in 2017 and eosinophil count.

All of the data were analysed using IBM SPSS 19.0 software (IBM, Armonk, NY, US).

### Performance and costs of the screening protocol

The following performance and economic indicators were calculated: yield of the screening, number needed to screen to detect one case (NNS), and cost per case detected. 

The definitions used for the performance and economic indicators were the following:

(i) Yield = number of NCC cases identified / total number of individuals screened ^×^ 100,000.

(ii) Number needed to screen to detect one case = total number screened / number of cases identified.

(iii) The costs of the screening algorithm were applied to the number of NCC cases diagnosed to give indicative costs per case of NCC detected: this was done by dividing the relative total cost of the tests in the algorithm by the number of NCC cases identified. To estimate the costs of the screening algorithm, we considered the costs of the services included: one (or two) serological tests for cysticercosis for all children and a brain MRI without contrast together with a clinical evaluation for all seropositive children as well as for seronegative children with compatible NCC signs/symptoms. Except for ELISAs (see below), the costs of these services were extracted from the ‘Catalogo Aziendale 2016’ of the Azienda Ospedaliero-Universitaria Careggi, Florence, Italy, which is a database reporting the list of standard set of medical services available in the Azienda Ospedaliero-Universitaria Careggi and their costs [32]. The costs of the considered services are similar in other Italian Regions. The cost of the cysticercosis ELISA serological test was extrapolated from the ‘Catalogo Regionale 2016’ of the Veneto Region [33].

This report was written according to the STrengthening the Reporting of OBservational studies in Epidemiology (STROBE) Statement guidelines.

## Results

The Figure reports the flow chart of screening. A total of 2,973 children who underwent a serological test for cysticercosis were included, 1,684 (56.6%) boys and 1,269 (42.7%) girls, while for 20 (0.7%) the sex was not available. These children accounted for ca 13% of the IAC from NCC endemic countries arriving in Italy in the study period (Supplement: Table 1). The median age was 6 years (interquartile range (IQR): 3–8). According to the origin in terms of geographical area, 1,271 (42.7%) were from Asia, 1,073 (36.1%) from Latin America/Caribbean, 516 (17.4%) from Africa, 27 (0.9%) from Europe, and for 86 (2.9%) the country of origin was unknown. An EITB was used to screen 2,437 (82.0%) of the children, including 2,389 with the test produced by LDBIO and 48 with the test produced by Immunetics. The ELISA test was employed to screen 1,534 (51.6%) adoptees. There were 998 (33.6%) individuals screened with both ELISA and EITB tests.

**Figure:**
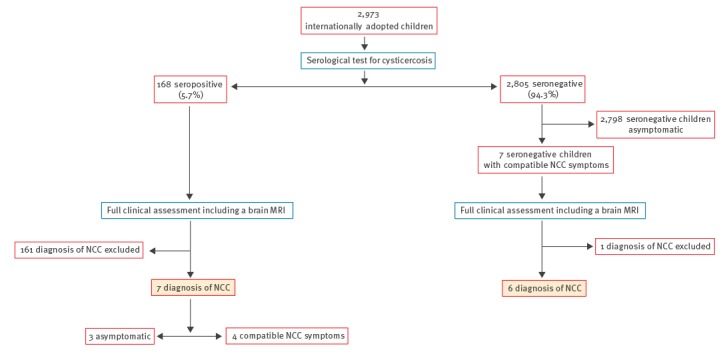
Flow chart of the screening process for cysticercosis in internationally adopted children, Italy, 2001–2016 (n = 2,973 children ≤16 years old screened)

MRI: magnetic resonance imaging; NCC: neurocysticercosis.

### Seroprevalence for cysticercosis

There were 168 (5.7%; 95% confidence interval (CI): 5.0–6.6) children seropositive to at least one test, 127 to ELISA only, 31 to an EITB only and 10 to both ELISA and EITB. The seroprevalence of cysticercosis with EITB (either LDBIO or Immunetics) interpreted according to the manufacturer was 1.7% (41/2,437; 95% CI: 1.2–2.2) while with ELISA it was 8.9% (137/1,534; 95% CI: 7.5–10.3). Table 2 reports in detail the seroprevalence according to the test used and the clinical centre. The seroprevalence in boys or girls was not significantly different, regardless of the laboratory test used to estimate it, i.e. EITB (either LDBIO or Immunetics, interpreted according to the manufacturer), or ELISA. Indeed, the seroprevalence obtained with EITB was 1.8% (26/1,417; 95% CI: 1.1–2.5) for boys and 1.5% (15/1,000; 95% CI: 0.8–2.2) for girls (p value = 0.6429). With ELISA this was 8.8% (75/856; 95% CI: 6.9–10.7) for boys and 9.1% (62/678; 95% CI: 6.9–11.3) for girls (p value = 0.8642).

**Table 2:** Estimations of seroprevalence for cysticercosis in internationally adopted children ≤16 years old according to the test used and the clinical centre, Italy, 2001–2016 (n = 2,973 children tested^a^)

Test	Meyer University Hospital^b^		Sacro Cuore Don Calabria Hospital^c^		All	
	n/N	%	n/N	%	n/N	%
EITB	10/1,315	0.8	31/1,122	2.8	41/2,437	1.7
ELISA	0/0	NA	137/1,534	8.9	137/1,534	8.9

EITB: enzyme-linked immune electro transfer blot; ELISA: enzyme-linked immunosorbent assay; n: number of individuals testing positive in serology for cysticercosis; N: total number of tested patients; NA: not applicable.

^a^ Some children were tested with only EITB, others with only ELISA, and some with both.

^b^ Infectious Diseases Unit, Meyer University Hospital, Florence, Italy.

^c^ Department of Paediatrics, Sacro Cuore Hospital-Don Calabria, Negrar, Italy.

The seroprevalence of cysticercosis according to the country and continent of origin of the children is reported in Table 3.

**Table 3:** Seroprevalence for cysticercosis and frequency of neurocysticercosis in internationally adopted children ≤16 years old according to the country and geographical area of birth, Italy, 2001–2016 (n = 2,973 children)

Geographical area and country of birth	EITB		ELISA		Frequency of NCC	
	n^a^/N	%	n^a^/N	%	n^b^/N	%
**AFRICA**	**9/463**	**1.9**	**9/151**	**6.0**	**4/522**	**0.8**
Benin	0/1	0.0	0/0	0.0	0/1	0.0
Burkina Faso	1/39	2.6	0/3	0.0	1/39	2.6
Cameroon	0/1	0.0	0/1	0.0	0/1	0.0
Cape Verde	0/1	0.0	0/0	0.0	0/1	0.0
Côte d’Ivoire	0/3	0.0	0/0	0.0	0/3	0.0
Eritrea	0/4	0.0	0/0	0.0	0/4	0.0
Ethiopia	6/301	2.0	9/128	7.0	0/353	0.0
The Gambia	0/1	0.0	0/0	0.0	0/1	0.0
Ghana	0/0	0.0	0/3	0.0	0/3	0.0
Guinea-Bissau	0/3	0.0	0/1	0.0	1/3	33.3
Kenya	0/17	0.0	0/2	0.0	0/17	0.0
Madagascar	0/4	0.0	0/2	0.0	0/6	0.0
Mali	0/8	0.0	0/0	0.0	0/8	0.0
Morocco	0/5	0.0	0/2	0.0	0/6	0.0
Nigeria	0/2	0.0	0/0	0.0	0/2	0.0
Democratic Republic of the Congo	1/63	1.6	0/8	0.0	2/63	3.2
Senegal	0/2	0.0	0/0	0.0	0/2	0.0
Somalia	0/2	0.0	0/0	0.0	0/2	0.0
Uganda	0/0	0.0	0/1	0.0	0/1	0.0
Unspecified country	1/6	16.6	0/0	0.0	0/6	0.0
**ASIA**	**22/889**	**2.5**	**93/854**	**10.9**	**6/1,271**	**0.5**
Armenia	0/2	0.0	0/0	0.0	0/2	0.0
Bangladesh	0/3	0.0	0/3	0.0	0/4	0.0
Cambodia	0/89	0.0	4/59	6.8	0/97	0.0
China	0/37	0.0	0/26	0.0	0/37	0.0
Philippines	0/34	0.0	0/6	0.0	0/34	0.0
India	20/530	3.8	83/633	13.1	4/870	0.5
Kazakhstan	0/3	0.0	0/0	0.0	0/3	0.0
Nepal	2/74	2.7	2/68	2.9	1/94	1.1
Pakistan	0/4	0.0	1/4	25.0	0/4	0.0
Sri Lanka	0/17	0.0	0/5	0.0	0/17	0.0
Thailand	0/7	0.0	0/2	0.0	0/7	0.0
Vietnam	0/87	0.0	2/37	5.4	1/91	1.1
Unspecified country	0/2	0.0	1/11	9.10	0/11	0.0
**LATIN AMERICA/CARIBBEAN**	**10/1,016**	**1.0**	**32/465**	**6.9**	**3/1,073**	**0.3**
Argentina	0/1	0.0	0/0	0.0	0/1	0.0
Bolivia	1/81	1.2	3/53	5.7	0/91	0.0
Brazil	4/248	1.6	9/112	8.0	1/269	0.4
Chile	1/86	1.2	1/17	5.9	0/87	0.0
Colombia	2/383	0.5	13/188	6.9	2/397	0.5
Costa Rica	1/26	3.8	0/2	0.0	0/26	0.0
Ecuador	0/14	0.0	0/16	0.0	0/17	0.0
El Salvador	0/1	0.0	0/0	0.0	0/1	0.0
Guatemala	0/4	0.0	0/1	0.0	0/4	0.0
Haiti	0/12	0.0	0/4	0.0	0/12	0.0
Honduras	0/4	0.0	0/1	0.0	0/4	0.0
Mexico	0/4	0.0	0/3	0.0	0/6	0.0
Nicaragua	0/1	0.0	0/0	0.0	0/1	0.0
Peru	1/147	0.7	6/65	9.2	0/152	0.0
Dominican Republic	0/4	0.0	0/2	0.0	0/4	0.0
Unspecified country	0/0	0.0	0/1	0.0	0/1	0.0
**EUROPE**	**0/22**	**0.0**	**1/7**	**14.3**	**0/27**	**0.0**
Albania	0/1	0.0	0/1	0.0	0/2	0.0
Bosnia and Herzegovina	0/0	0.0	0/1	0.0	0/1	0.0
Bulgaria	0/4	0.0	0/0	0.0	0/4	0.0
Latvia	0/1	0.0	0/0	0.0	0/1	0.0
Lithuania	0/3	0.0	0/0	0.0	0/3	0.0
Moldova	0/2	0.0	0/0	0.0	0/2	0.0
Poland	0/2	0.0	0/0	0.0	0/2	0.0
Russia^c^	0/7	0.0	0/2	0.0	0/7	0.0
Serbia	0/1	0.0	0/0	0.0	0/1	0.0
Ukraine	0/1	0.0	1/3	33.3	0/4	0.0
**Unspecified origin**	0/**47**	**0.0**	**2/57**	**3.5**	**0/80**	**0.0**

EITB: enzyme-linked Immune electro transfer blot; ELISA: enzyme-linked immunosorbent assay; N: total number of patients tested with a serological test for cysticercosis; NCC: neurocysticercosis.

^a^ Number of individuals testing positive in a serological test for cysticercosis.

^b^ Number of children diagnosed with neurocysticercosis according to the criteria proposed by Del Brutto et al. in 2017 (probable or definitive diagnosis).

^c^ European part of Russia.

### Frequency of neurocysticercosis according to the 2017 version of Del Brutto et al. criteria

NCC was diagnosed according to the 2017 version of Del Brutto et al. criteria in 13 children accounting for a frequency of 0.4% (13/2,973; 95% CI: 0.2–0.6) in the study population. According to these criteria, 10 were diagnosed as definitive cases and three as probable cases (Supplement: Table 3). Among 13 children with a diagnosis of NCC (2017 version of Del Brutto et al. criteria), seven were seropositive to at least a serological test for cysticercosis (five were positive to EITB only, one to both EITB and ELISA, one to ELISA only). NCC was diagnosed in 4.2% of all seropositive children (7/168). Among children with a diagnosis of NCC, six of 13 were seronegative for cysticercosis. These children presented with neurological symptoms (epilepsy) that lead to the diagnosis of NCC following a full clinical assessment, which included at least a brain MRI. Of seropositive children with NCC, three were asymptomatic and four symptomatic (epilepsy). Nine of the 10 symptomatic children with NCC (four seropositive and six seronegative) had active or degenerating NCC lesions at neuroimaging, while one child had only calcified lesions. Among the three asymptomatic seropositive individuals, all three had active or degenerating lesions. According to the type of brain lesions, seven children had a single brain lesion each (all enhancing lesions; three seropositive, four seronegative), one had two lesions (cysts without scolex; seropositive), four had three or more enhancing lesions (three seropositive, one seronegative), one had calcification only (seronegative). Children with active or degenerating lesions were treated with albendazole and corticosteroids. Individuals with epilepsy were treated with antiepileptic drugs.

The frequency of NCC in boys and girls was not significantly different (0.5% (8/1,684; 95% CI: 0.2–0.8) and 0.4% (5/1,269; 95% CI: 0.1–0.7), respectively, p value = 0.7419). The median age of children with and without NCC was not significantly different: 6 and 6 years respectively (p value = 0.6324). The prevalence of eosinophilia (defined as eosinophil count > 450 per µL) among individuals with available data was not statistically different among patients with NCC (4/13, 30.8%; 95% CI: 5.7–55.9) and those without NCC (446/933, 47.8%; 95% CI: 44.6–51) (p value = 0.3464).

The frequencies of NCC according to the country and geographical area of origin of the children are reported in Table 3.

Sensitivity, specificity, PPV and NPV of EITB and ELISA considering as gold standard the diagnosis of NCC (probable and definitive diagnosis, according to the 2017 diagnostic criteria by Del Brutto et al.) are reported in Table 4.

**Table 4:** Sensitivity, specificity, positive predictive value and negative predictive value of EITB and ELISA cysticercosis tests, for the diagnosis of neurocysticercosis^a^ in internationally adopted children ≤16 years old, Italy, 2001–2016

Test	Sensitivity		Specificity		PPV (%)	NPV (%)
	n^b^/N^c^	%	n^d^/N^e^	%		
EITB	6/11^a^	54.5	2,391/2,426	98.6	14.6	99.8
ELISA	2/9^a^	22.2	1,390/1,525	91.1	1.4	99.5

EITB: enzyme-linked immune electro transfer blot; ELISA: enzyme-linked immunosorbent assay; NCC: neurocysticercosis; NPV: negative predictive value; PPV: positive predictive value.

^a^ Among the 13 patients with a diagnosis of NCC, all were tested with at least one serological test: seven with both ELISA and EITB, two with ELISA only and four with EITB only. In total, 11 were tested with EITB and nine with ELISA. The diagnosis of NCC was according to the diagnostic criteria proposed by Del Brutto et al. in 2017 (probable and definitive diagnosis).

^b^ Number of NCC-diagnosed children who were respectively found seropositive for cysticercosis by EITB or ELISA.

^c^ Number of NCC-diagnosed children who were respectively serologically tested for cysticercosis by EITB or ELISA.

^d^ Number of children who tested seronegative for cysticercosis either by EITB or ELISA, excluding those with a diagnosis of NCC.

^e^ Number of children who were serologically tested for cysticercosis by either EITB or ELISA, excluding those with a diagnosis of NCC.

The associations between EITB and ELISA positivity and eosinophilia and the diagnosis of other helminthic infection are reported in Table 5. Noteworthy, there was a statistically significant correlation between infection due to *Hymenolepis nana* and positivity of either EITBs or ELISA. Of the 1,638 children undergoing parasitological stool examination, the following helminthic infections were diagnosed: *H. nana* (n = 285; 17.4%) *Trichuris trichiura* (n = 44), hookworms (n = 17), *Schistosoma* spp. (n = 12), *Strongyloides stercoralis* (n = 8), *Enterobius vermicularis* (n = 4), *Taenia saginata* (n = 3) and *T. solium* (n = 1).

**Table 5:** Association between seropositivity for cysticercosis and presence of other helminthic infection or eosinophilia in internationally adopted children ≤16 years old, Italy, 2001–2016

Other helminthic infection or eosinophilia	Positive EITB for cysticercosis		Negative EITB for cysticercosis		OR (CI)	p value
	n^a^/N^b^	%	n^c^/N^d^	%		
*Hymenolepis nana* on stool^i^	12/37	32.4	147/1,120	13.1	3.18 (1.56–6.6)	0.0008
Other helminthic infections^j^	1/37	2.7	37/1,120	3.3	0.81 (0.11–6.09)	0.8401
Eosinophilia^k^	11/26	42.3	278/641	43.3	0.96 (0.43–2.12)	0.9147
**Other helminthic infection or eosinophilia**	**Positive ELISA for cysticercosis**		**Negative ELISA for cysticercosis**		OR **(CI)**	**p value **
	**n^e^/N^f^**	**%**	**n^g^/N^h^**	**%**		
*Hymenolepis nana* on stool^i^	73/133	54.9	200/1,099	18.2	5.47 (3.76–7.95)	< 0.0001
Other helminthic infections^j^	12/133	9.0	72/1,099	6.5	1.41 (0.75–2.68)	0.2856
Eosinophilia^k^	74/107	69.2	366/805	45.5	2.69 (1.74–4.15)	< 0.00001

CI: confidence interval; EITB: enzyme-linked immune electro transfer blot; ELISA: enzyme-linked immunosorbent assay; OR: odds ratio.

^a^ Number of individuals with the listed feature found seropositive with EITB.

^b^ Number of individuals found seropositive with EITB and with available information on the listed feature.

^c^ Number of individuals found seronegative with EITB and with the listed feature.

^d^ Number of individuals found seronegative with EITB negative and with available information on the listed feature.

^e^ Number of individuals found seropositive with ELISA and with the listed feature.

^f^ Number of individuals found seropositive with ELISA and with available information on the listed feature.

^g^ Number of individuals found seronegative with ELISA and with the listed feature.

^h^ Number of individuals found seronegative with ELISA and with available information on the listed feature.

^i^ Presence of *Hymenolepis nana* at the parasitological examination of stool.

^j^ Diagnosis of any other helminthic infection through parasitological stool test.

^k^ Eosinophils count > 450 per µL.

Performance and costs of the screening protocol: the yield of the screening programme was 437/100,000, in our population. The NNS was 228. The cost of the screening was EUR 134,831 and EUR 8,426.9 for the 16 years period and per year in mean respectively and the cost per case detected was EUR 10,372 (Table 6).

**Table 6:** Costs of services included in the screening algorithm for neurocysticercosis in internationally adopted children ≤16 years old, Italy, 2001–2016

Service	Number of services used	Unitary cost in euros	Total cost in euros
ELISA serological test^a^	1,534	8.8	13,499
EITB serological test^b^	2,437	36	87,732
Brain MRI without contrast^b^	175	170	29,750
Infectious diseases’ consultation^b^	175	22	3,850
The entire screening programme			134,831

EITB: enzyme-linked immune electro transfer blot; ELISA: enzyme-linked immunosorbent assay; MRI: magnetic resonance imaging.

^a^ Cost of the service obtained from ‘Catalogo Regionale 2016’ of the Veneto Region, Italy [33].

^b^ Cost of the service obtained from ‘Catalogo Aziendale 2016’ of the Azienda Ospedaliero-Universitaria Careggi, Florence, Italy [32].

## Discussion

The present study reveals a substantial seroprevalence of cysticercosis in IAC ranging between 1.7% and 8.9% depending on the test used (EITB and ELISA, respectively), while the frequency of NCC was 0.4%. According to the geographical area of origin, the frequency of NCC was higher in IAC from Africa (0.8%) compared with Asia (0.5%), Latin America/Caribbean (0.3%) and Europe (0%). This finding confirms that, even if cysticerocosis/*T. solium* taeniasis has been considered for a long time endemic mainly in Latin America and South-East Asia, the disease is present also in large areas of Sub-Saharan Africa, as suggested by accumulating evidence [4,34]. Of note, this result does not support the most recent version of the Italian protocol of the ‘National Working Group for the Migrant Child’ of the Italian Society of Paediatrics [25] to exclude IAC from Africa among children screened from NCC.

There are few smaller previous studies investigating the performance of a serological screening for cysticercosis and NCC in IAC. In a French study, carried out by Blanchi et al., only IAC from Haiti and Madagascar (considered at high risk) were serologically screened for cysticercosis. In this study, only one of 25 Haitian children was seropositive (the authors did not specify whether the patient was affected by NCC or cysticercosis) [27]. According to a study conducted in Italy by Valentini et al., children from Asia, Africa and Latin America were serologically screened for cysticercosis with an ELISA test, and those testing positive were tested with an EITB for confirmation. Thirty-two of 358 (8.9%) children were seropositive by ELISA, but none by EITB [22]. The authors of these two previous studies reached opposite conclusions. Blanchi et al. suggested that serological screening of asymptomatic IAC from highly endemic countries for cysticercosis should be mandatory, since it can help to detect asymptomatic patients with lesions allowing their treatment before the symptoms appear. On the contrary, Valentini et al. suggested that the screening in asymptomatic IAC could be avoided given the absence of EITB confirmed positive results.

In the majority of cases, patients with NCC develop symptoms some years after the infection occurred. For example, according to a systematic review of the literature, 73.6% of patients with imported NCC in Europe developed symptoms 2–5 years after being exposed in an endemic country [3] and classical epidemiological studies carried out in the 1960s in English soldiers returning from India obtained very similar results [35]. For these reasons, screening for cysticercosis in asymptomatic individuals could be theoretically useful in people at risk for NCC to early detect infected subjects, treat them and prevent the onset of symptoms. However, there is no practical evidence in favour of screening of asymptomatic people [36]. From the scarce information on natural evolution of cysticercosis, most infected persons will never develop symptoms [37]. For example in an endemic rural area of Peru the prevalence of NCC according to computed tomography (CT) scan (which was offered to all > 18 year-old villagers) was 18.8%, but among individuals with NCC only 17% reported a history of epilepsy or headache [37].

In symptomatic patients with NCC, growing evidence has cumulated about the efficacy of antiparasitic treatment (combined with corticosteroids) in reducing the number of brain cysts and the risk of seizure recurrence after treatment [38-44]. However, there is no evidence that treating an asymptomatic person harbouring a live parasite will reduce the probability for neurological symptoms in the future and to what extent.

Recently, a multidisciplinary study group on cysticercosis did not recommend serological screening for cysticercosis in asymptomatic subjects [45]. Instead, the group recommended a careful clinical assessment to investigate unrecognised compatible symptoms of cysticercosis (a history of convulsions and/or other compatible neurological signs or symptoms) and a physical examination to detect subcutaneous nodules compatible with cysticercosis in persons at high risk for cysticercosis. Those considered at high risk were patients with confirmed *T. solium* taeniasis, household and daily contacts of confirmed *T. solium* taeniasis cases, family members of cysticercosis cases who are likely to have been exposed to the same environment as the index case. The same approach was recommended for individuals with possible exposure in an endemic country before undergoing treatment with antiparasitic drugs, such as praziquantel and albendazole, which may cause adverse reactions in case of unrecognised cysticercosis. Moreover, individuals with compatible signs or symptoms should be fully assessed including appropriate brain imaging (CT scan and/or MRI) [45]. The utility of a serological screening for cysticercosis was previously debated by other author groups [36], while the US Centers for Disease Control and Prevention (CDC) recommends a clinical assessment for cysticercosis before prescribing albendazole or praziquantel to refugees in order to avoid adverse reactions such as seizures in subjects with unrecognised NCC [46].

Our data showed several limitations of serological screening for cysticercosis. Firstly, the sensitivity for the diagnosis of NCC is low, both for EITB and ELISA test (54.5% and 22.2% respectively), with six of 13 children with NCC being seronegative. The low sensitivity of serological tests can be explained mainly by the known low sensitivity (< 50–60%) of serological tests, including EITB, in patients with a single brain lesion [47] who are the majority of patients in this study (n = 7). This was an expected finding, considering that a single enhancing lesion is the most common form of NCC in children [48].

Moreover, it should be noted that the majority of subjects with NCC (10 of 13) were symptomatic for epilepsy and they would be probably diagnosed with NCC even without the serological screening. However, three of 13 children with NCC were asymptomatic and would not have been identified in the absence of serological screening.

The high rate of individuals with a serological test positive for cysticercosis but without a diagnosis of NCC may be at least partially explained by possible false positivity (cross-reaction) of the test in case of infection caused by *H. nana*. Intestinal infection with *H. nana* is a quite frequent finding in IAC and the reported prevalence is 1% [49], 2% [50], 9.6% [22], according to the different case series. In the present study, the prevalence of *H. nana* infection was 17.4%. Co-infection with this parasite is therefore common in IAC and adds a further limitation to the effectiveness of the serological screening for cysticercosis.

Finally, the cost of the screening, EUR 10,372 per case, is not negligible, and much higher when compared with the reported cost for other infections targeted by different screening protocols, such as latent tuberculosis infection [51,52].

Although the epidemiological pattern of IAC in different high income countries can change, we think the results of our study have a good generalisability. Although limited by retrospective nature, this is a wide study, with high representation of the different endemic parts of the world. However, data concerning the economic analysis are based on the current Italian system and therefore have a limited external validity.

Another limitation of the study was the inclusion of some (n = 27) Eastern European children, screened, not systematically, for cysticercosis, despite the protocol of the ‘National Working Group for the Migrant Child’ not recommending the screening of children of European origin. The reasons why the clinician in charge decided to prescribe the screening are unknown (probably eosinophilia). However, we think that the inclusion of these children in the analysis has not affected the results and our conclusions, since the Eastern European children screened were few, only one individual was seropositive with ELISA, and no case of NCC were diagnosed. Moreover, the choice of using the Del Brutto et al. criteria as gold standard for the diagnosis of NCC negatively influenced the performance of the ELISA test compared with the EITB since only the latter is considered among the diagnostic criteria proposed by the authors. However the poor performance of ELISA for the diagnosis of NCC has been largely demonstrated [16,53-57].

Finally, the reliability of specificity and NPV of the two serological tests is limited since the MRI was not made in seronegative subjects.

In conclusion, NCC is rare in IAC and serological screening for NCC presents several challenges, such as the poor performance of laboratory test, possible cross-reaction with other infections, the high cost, the unknown benefit of treating subjects with active NCC lesions in absence of symptoms. We propose that the ongoing serological screening for cysticercosis should be discontinued, at least in Italy, unless new evidence in support will become available in the future.

## References

[1] Garcia HH Nash TE Del Brutto OH Clinical symptoms, diagnosis, and treatment of neurocysticercosis. Lancet Neurol. 2014;13(12):1202-15. 10.1016/S1474-4422(14)70094-8 25453460 PMC6108081

[2] Cantey PT Coyle CM Sorvillo FJ Wilkins PP Starr MC Nash TE Neglected parasitic infections in the United States: cysticercosis. Am J Trop Med Hyg. 2014;90(5):805-9. 10.4269/ajtmh.13-0724 24808248 PMC4015568

[3] Zammarchi L Strohmeyer M Bartalesi F Bruno E Muñoz J Buonfrate D Epidemiology and management of cysticercosis and Taenia solium taeniasis in Europe, systematic review 1990-2011. PLoS One. 2013;8(7):e69537. 10.1371/journal.pone.0069537 23922733 PMC3726635

[4] World Health Oranization (WHO). Fourth WHO report on Neglected Tropical Diseases. Integrating Neglected Tropical Diseases into Global Health and Development. Geneva: WHO; 2017.

[5] Laranjo-González M Devleesschauwer B Trevisan C Allepuz A Sotiraki S Abraham A Epidemiology of taeniosis/cysticercosis in Europe, a systematic review: Western Europe. Parasit Vectors. 2017;10(1):349. 10.1186/s13071-017-2280-8 28732550 PMC5521153

[6] Willingham AL Engels D Control of Taenia solium cysticercosis/taeniosis. Adv Parasitol. 2006;61:509-66. 10.1016/S0065-308X(05)61012-3 16735172

[7] Ndimubanzi PC Carabin H Budke CM Nguyen H Qian YJ Rainwater E A systematic review of the frequency of neurocyticercosis with a focus on people with epilepsy. PLoS Negl Trop Dis. 2010;4(11):e870. 10.1371/journal.pntd.0000870 21072231 PMC2970544

[8] Bruno E Bartoloni A Zammarchi L Strohmeyer M Bartalesi F Bustos JA Epilepsy and neurocysticercosis in Latin America: a systematic review and meta-analysis. PLoS Negl Trop Dis. 2013;7(10):e2480. 10.1371/journal.pntd.0002480 24205415 PMC3814340

[9] Del Brutto OH Neurocysticercosis in infants and toddlers: report of seven cases and review of published patients. Pediatr Neurol. 2013;48(6):432-5. 10.1016/j.pediatrneurol.2013.02.001 23668866

[10] Fleury A Morales J Bobes RJ Dumas M Yánez O Piña J An epidemiological study of familial neurocysticercosis in an endemic Mexican community. Trans R Soc Trop Med Hyg. 2006;100(6):551-8. 10.1016/j.trstmh.2005.08.008 16316671

[11] Prasad KN Verma A Srivastava S Gupta RK Pandey CM Paliwal VK An epidemiological study of asymptomatic neurocysticercosis in a pig farming community in northern India. Trans R Soc Trop Med Hyg. 2011;105(9):531-6. 10.1016/j.trstmh.2011.06.001 21764415

[12] Kumar A Mandal A Sinha S Singh A Das RR Prevalence, Response to Cysticidal Therapy, and Risk Factors for Persistent Seizure in Indian Children with Neurocysticercosis. Int J Pediatr. 2017;2017:8983958. 10.1155/2017/8983958 28167968 PMC5259654

[13] Banerjee TK Hazra A Biswas A Ray J Roy T Raut DK Neurological disorders in children and adolescents. Indian J Pediatr. 2009;76(2):139-46. 10.1007/s12098-008-0226-z 19082533

[14] Gaffo AL Guillén-Pinto D Campos-Olazábal P Burneo JG Cysticercosis as the main cause of partial seizures in children in Peru. Rev Neurol. 2004;39(10):924-6. 15573306

[15] Townes JM Hoffmann CJ Kohn MA Neurocysticercosis in Oregon, 1995-2000. Emerg Infect Dis. 2004;10(3):508-10. 10.3201/eid1003.030542 15109424 PMC3322801

[16] Zammarchi L Angheben A Gobbi F Zavarise G Requena-Mendez A Marchese V Profile of adult and pediatric neurocysticercosis cases observed in five Southern European centers. Neurol Sci. 2016;37(8):1349-55. 10.1007/s10072-016-2606-x 27193586 PMC4956690

[17] Commissione per le Adozioni Internazionali (CAI) [International Adoption Committee]. L'Italia in controtendenza rispetto al calo delle adozioni internazionali di tutti gli altri Paesi di accoglienza. [Italy in contrast with the decline in international adoptions of all other host countries]. Rome: CAI; 2016. Italian. Available from: http:// http://www.commissioneadozioni.it/it/notizie/2016/dati-adozioni.aspx

[18] Hénaff F Hazart I Picherot G Baqué F Gras-Le Guen C Launay E Frequency and characteristics of infectious diseases in internationally adopted children: a retrospective study in Nantes from 2010 to 2012. J Travel Med. 2015;22(3):179-85. 10.1111/jtm.12196 25787709

[19] Eckerle JK Howard CR John CC Infections in internationally adopted children. Pediatr Clin North Am. 2013;60(2):487-505. 10.1016/j.pcl.2012.12.010 23481113

[20] Sollai S Ghetti F Bianchi L de Martino M Galli L Chiappini E Infectious diseases prevalence, vaccination coverage, and diagnostic challenges in a population of internationally adopted children referred to a Tertiary Care Children’s Hospital from 2009 to 2015. Medicine (Baltimore). 2017;96(12):e6300. 10.1097/MD.0000000000006300 28328809 PMC5371446

[21] Chiappini E Sollai S de Martino M Galli L Malaria in Children Adopted from the Democratic Republic of the Congo. Emerg Infect Dis. 2017;23(4):721-2. 10.3201/eid2304.161777 28322706 PMC5367411

[22] Valentini P Gargiullo L Ceccarelli M Ranno O Health status of internationally adopted children. The experience of an Italian "GLNBI" paediatric centre. Ital J Public Health. 2012;9:e7527-32.

[23] Gruppo di Lavoro Nazionale per il Bambino Migrante della Società Italiana di Pediatria. [National Working Group for the Migrant Child of the Italian Society of Paediatrics]. Italy: Società Italiana di Pediatria [Italian Society of Paediatrics]. Italian. Available from: http://www.glnbi.org/

[24] Adami Lami C, Gabrielli C, Zaffaroni M, Cataldo F, Valentini F, et al. (2007) Nuovo Protocollo 2007 per l'accoglienza sanitaria del bambino adottato all'estero. [New Protocol 2007 for health-reception of internationally adopted children]. Atti Congresso della Società Italiana di Pediatria, 2007.[Proceedings of the Congress of the Italian Society of Paediatrics]. Italian. Available from: http://www.glnbi.org/documenti/ad88407a4ca3bff42fa5666d67028df0.pdf

[25] Zavarise G. Protocollo del "Gruppo di Lavoro Nazionale per il Bambino Migrante" (GLNBI) per il bambino adottato 2013 Bozza proposta per revisione del protocollo per l'accoglienza sanitaria del bambino adottato all'estero. [Protocol of the "National Working Group for the Migrant Child" for the adopted children. Draft proposal for the revision of the protocol for health-reception for internationally adopted children 2013]. Riunione del Gruppo di Lavoro Nazionale per il Bambino Migrante della Società Italiana di Pediatria [Meeting of the National Working Group for the Migrant Child of the Italian Society of Paediatrics], Bologna 9 maggio 2013. Italian. Available from: http://www.glnbi.org/documenti/869858a7d1b145d4f04e3c368e15f8df.pdf

[26] Protocollo diagnostico-assistenziale per i bambini adottati all’estero. [Protocol for diagnosis and management of internationally adopted children]. In: Atti del 58° Congresso della Società Italiana di Pediatria. [Proceedings of the 58^th^ Congress of the Italian Society of Paediatrics]; 2002 30 Sep-2 Oct.; Montecatini, Italy. Italian

[27] Blanchi S Chabasse D Pichard E Darviot E de Gentile L Post-international adoption medical follow-up at the Angers university hospital between 2009 and 2012. Med Mal Infect. 2014;44(2):69-75. 10.1016/j.medmal.2013.12.003 24486252

[28] Del Brutto OH Nash TE White AC Jr Rajshekhar V Wilkins PP Singh G Revised diagnostic criteria for neurocysticercosis. J Neurol Sci. 2017;372:202-10. 10.1016/j.jns.2016.11.045 28017213

[29] LDBIO Diagnostics (2016) Cysticercosis Western Blot IgG. Instruction for use.

[30] DRG International Inc USA (2011) Enzyme Linked Immune Assay, Taenia Solium IgG. Package insert.

[31] QualicodeTM Cysticercosis kit (Immunetics Inc., Boston, Massachusetts, USA. Package Insert. [Accessed 21 Aug 2017]. Available from: https://wwwyumpucom/en/document/view/26499278/qualicodea-cysticercosis-kit-invitech

[32] Catalogo Aziendale (ex nomenclatore) 2006. [Organisation’s catalogue 2006]. Florence Italy: Azienda Ospedaliero Universitaria Careggi; 2016.

[33] Catalogo Regionale Prescivibile. [Prescivable Regional Catalogue]. Regione del Veneto; 2016.

[34] Phiri IK Ngowi H Afonso S Matenga E Boa M Mukaratirwa S The emergence of Taenia solium cysticercosis in Eastern and Southern Africa as a serious agricultural problem and public health risk. Acta Trop. 2003;87(1):13-23. 10.1016/S0001-706X(03)00051-2 12781374

[35] Dixon H Lipscomb F Cysticercosis: An analysis and follow up of 450 cases. Medical Research Council Special Report Series, London. 1961;299:1-58.

[36] Garcia HH Rodriguez S Gilman RH Gonzalez AE Tsang VC Cysticercosis Working Group in Peru Neurocysticercosis: is serology useful in the absence of brain imaging? Trop Med Int Health. 2012;17(8):1014-8. 10.1111/j.1365-3156.2012.03037.x 22809375

[37] Moyano LM O’Neal SE Ayvar V Gonzalvez G Gamboa R Vilchez P High Prevalence of Asymptomatic Neurocysticercosis in an Endemic Rural Community in Peru. PLoS Negl Trop Dis. 2016;10(12):e0005130. 10.1371/journal.pntd.0005130 27992429 PMC5167259

[38] Garcia HH Del Brutto OH Cysticercosis Working Group in Peru Antiparasitic treatment of neurocysticercosis - The effect of cyst destruction in seizure evolution. Epilepsy Behav. 2017;76:158-62. 10.1016/j.yebeh.2017.03.013 28606690 PMC5675823

[39] Baranwal AK Singhi PD Khandelwal N Singhi SC Albendazole therapy in children with focal seizures and single small enhancing computerized tomographic lesions: a randomized, placebo-controlled, double blind trial. Pediatr Infect Dis J. 1998;17(8):696-700. 10.1097/00006454-199808000-00007 9726343

[40] Carpio A Kelvin EA Bagiella E Leslie D Leon P Andrews H Effects of albendazole treatment on neurocysticercosis: a randomised controlled trial. J Neurol Neurosurg Psychiatry. 2008;79(9):1050-5. 10.1136/jnnp.2008.144899 18495737

[41] Garcia HH Pretell EJ Gilman RH Martinez SM Moulton LH Del Brutto OH A trial of antiparasitic treatment to reduce the rate of seizures due to cerebral cysticercosis. N Engl J Med. 2004;350(3):249-58. 10.1056/NEJMoa031294 14724304

[42] Gogia S Talukdar B Choudhury V Arora BS Neurocysticercosis in children: clinical findings and response to albendazole therapy in a randomized, double-blind, placebo-controlled trial in newly diagnosed cases. Trans R Soc Trop Med Hyg. 2003;97(4):416-21. 10.1016/S0035-9203(03)90075-7 15259471

[43] Kalra V Dua T Kumar V Efficacy of albendazole and short-course dexamethasone treatment in children with 1 or 2 ring-enhancing lesions of neurocysticercosis: a randomized controlled trial. J Pediatr. 2003;143(1):111-4. 10.1016/S0022-3476(03)00211-7 12915835

[44] Singhi P Jain V Khandelwal N Corticosteroids versus albendazole for treatment of single small enhancing computed tomographic lesions in children with neurocysticercosis. J Child Neurol. 2004;19(5):323-7. 10.1177/088307380401900503 15224704

[45] Zammarchi L Bonati M Strohmeyer M Albonico M Requena-Méndez A Bisoffi Z Screening, diagnosis and management of human cysticercosis and Taenia solium taeniasis: technical recommendations by the COHEMI project study group. Trop Med Int Health. 2017;22(7):881-94. 10.1111/tmi.12887 28449318

[46] Centers for Disease Control and Prevention (CDC). Intestinal parasite guidelines for domestic medical examination for newly arrived refugees. Atlanta: CDC; 2013. Available from: https://www.cdc.gov/immigrantrefugeehealth/pdf/intestinal-parasites-domestic.pdf

[47] Rajshekhar V Oommen A Serological studies using ELISA and EITB in patients with solitary cysticercus granuloma and seizures. Neurol Infect Epidemiol. 1997;2:177-80.

[48] Singhi P Ray M Singhi S Khandelwal N Clinical spectrum of 500 children with neurocysticercosis and response to albendazole therapy. J Child Neurol. 2000;15(4):207-13. 10.1177/088307380001500401 10805184

[49] Staat MA Rice M Donauer S Mukkada S Holloway M Cassedy A Intestinal parasite screening in internationally adopted children: importance of multiple stool specimens. Pediatrics. 2011;128(3):e613-22. 21824880 10.1542/peds.2010-3032 PMC9923786

[50] Sarfaty M Rosenberg Z Siegel J Levin RM Intestinal parasites in immigrant children from Central America. West J Med. 1983;139(3):329-31. 6636747 PMC1021515

[51] Zammarchi L Casadei G Strohmeyer M Bartalesi F Liendo C Matteelli A A scoping review of cost-effectiveness of screening and treatment for latent tubercolosis infection in migrants from high-incidence countries. BMC Health Serv Res. 2015;15(1):412. 10.1186/s12913-015-1045-3 26399233 PMC4581517

[52] Hardy AB Varma R Collyns T Moffitt SJ Mullarkey C Watson JP Cost-effectiveness of the NICE guidelines for screening for latent tuberculosis infection: the QuantiFERON-TB Gold IGRA alone is more cost-effective for immigrants from high burden countries. Thorax. 2010;65(2):178-80. 10.1136/thx.2009.119677 19996345

[53] Garcia HH Castillo Y Gonzales I Bustos JA Saavedra H Jacob L Low sensitivity and frequent cross-reactions in commercially available antibody detection ELISA assays for Taenia solium cysticercosis. Trop Med Int Health. 2018;23(1):101-5. 10.1111/tmi.13010 29160912 PMC5760338

[54] Carod JF Randrianarison M Razafimahefa J Ramahefarisoa RM Rakotondrazaka M Debruyne M Evaluation of the performance of 5 commercialized enzyme immunoassays for the detection of Taenia solium antibodies and for the diagnosis of neurocysticercosis. Diagn Microbiol Infect Dis. 2012;72(1):85-9. 10.1016/j.diagmicrobio.2011.09.014 22085773

[55] Carvalho Junior RM Costa DL Soares SC Costa CH Evaluation of an enzyme immunoassay for clinical diagnosis of neurocysticercosis in symptomatic patients. Rev Soc Bras Med Trop. 2010;43(6):647-50. 10.1590/S0037-86822010000600009 21181016

[56] Chuang C Xing-Wang C Jian-Zhong D Tiao-Ying L [Evaluation of ELISA kit for detection of serum specific IgG antibodies against *Taenia solium* in diagnosis of human cysticercosis]. Zhongguo Xue Xi Chong Bing Fang Zhi Za Zhi. 2017;29(2):228-30. 29469334 10.16250/j.32.1374.2016216

[57] Das A Goyal R Saxena S Singh NP Diagnosis of neurocysticercosis by enzyme-linked immunosorbent assay. J Indian Med Assoc. 2005;103(10):528-9. 16498754

